# Enhanced transformation of incidentally learned knowledge into explicit memory by dopaminergic modulation

**DOI:** 10.1371/journal.pone.0199013

**Published:** 2018-06-14

**Authors:** Mareike Clos, Tobias Sommer, Signe L. Schneider, Michael Rose

**Affiliations:** Department for Systems Neuroscience, University Medical Center Hamburg Eppendorf, Martinistr. 52, Hamburg, Germany; Universiteit Gent, BELGIUM

## Abstract

During incidental learning statistical regularities are extracted from the environment without the intention to learn. Acquired implicit memory of these regularities can affect behavior in the absence of awareness. However, conscious insight in the underlying regularities can also develop during learning. Such emergence of explicit memory is an important learning mechanism that is assumed to involve prediction errors in the striatum and to be dopamine-dependent. Here we directly tested this hypothesis by manipulating dopamine levels during incidental learning in a modified serial reaction time task (SRTT) featuring a hidden regular sequence of motor responses in a placebo-controlled between-group study. Awareness for the sequential regularity was subsequently assessed using cued generation and additionally verified using free recall. The results demonstrated that dopaminergic modulation nearly doubled the amount of explicit sequence knowledge emerged during learning in comparison to the placebo group. This strong effect clearly argues for a causal role of dopamine-dependent processing for the development of awareness for sequential regularities during learning.

## Introduction

In incidental learning, statistical regularities are extracted from the environment and stored in memory without intention or instruction to learn. For instance, infants acquire the statistical regularities governing language during development without being explicitly told these rules. Once these regularities have been learned implicitly, conscious awareness of the underlying pattern can develop. Explicit representation of the regularities underlying one’s behavior in a given context is especially advantageous in a complex and changing world, as such conscious insight allows for a more adaptive control of one’s behavior [1]. Therefore, the transformation from implicit to explicit knowledge is a key process in the development of higher cognitive functions such as language or reasoning, as these functions are mostly acquired incidentally through experience.

A previous fMRI study revealed a prominent role of the striatum for the emergence of explicit knowledge in an incidental sequential learning task in which the participants had to respond with button presses to the presentation of visual stimuli [2]. Importantly, the task featured a sequential regularity that the participants were naïve to in the beginning but could learn incidentally by performing the task. During learning, activity in the ventral striatum (VS) increased time-locked to the emergence of explicit knowledge which can be observed at the behavioral level. That is, the activation in the VS started to rise just before an abrupt drop in response times indicated that the participants had detected the hidden sequential regularity and thus could adapt their strategy to initiate the appropriate motor response even before the next stimuli appeared. Such emergence of explicit memory has been proposed to involve prediction errors, as the acceleration of responses during the incidental learning is unexpected for the participants. The detection of the discrepancy between expected and observed speed is then thought to trigger a search process which ultimately leads to conscious insight [3,4]. The relevance of the striatum for this presumed prediction error-based transformation from implicit to explicit memory is more generally in accordance with the striatum’s well-established role in prediction errors. Dopaminergic projections from the midbrain to the striatum are thought to signal prediction errors not only in reinforcement learning [5–7] but also in learning tasks without reward feedback [8–11]. Additional support for dopaminergic striatal processes in sequential regularity learning comes from a PET study demonstrating that explicit memory retrieval of learned sequences induces dopamine release in the striatum [12]. Furthermore, impairments in implicit sequence learning [13–15] and in retrieval of implicitly learned sequences [16] have been noted in patients with Parkinson’s Disease; these impairments were moreover affected by antiparkinsonian dopaminergic therapy [17,18]. Taken together, there is good evidence that dopaminergic processes in the striatum are relevant for the emergence of explicit memory for hidden structural regularities.

To directly test this hypothesis we here manipulated dopamine levels using 2 mg of the D2-receptor antagonist haloperidol in a randomized placebo-controlled between group design as part of a larger study [19] and assessed the emergence of explicit memory during incidental learning within a modified version of the classical serial reaction time task (SRTT) [20].

Despite the antagonistic effect in chronic treatment [21], acute low doses of D2-antagonists (which, in contrast to higher doses, do not evoke cataleptic side effects) are thought to preferably block presynaptic D2-autoreceptors due to their higher dopamine affinity [22] and therefore lead to increased dopamine release [23,24]. Indeed, various studies have demonstrated that acute administration of D2-antagonists boosts activity of dopamine neurons and dopamine release in the striatum [25–31] and increases striatal blood flow in both animals [25,32] and in humans [33]. We chose for a dose of 2 mg haloperidol, as doses in the range of 1–3 mg had been administered in previous human studies without occurrence of obvious side effects (e.g., [23,34–37] and thus ruling out significant postsynaptic blockade effects. Moreover, doses of 0.03 mg/kg in rats (corresponding to 2.1 mg for 70 kg) were associated with *increased* locomotion behaviour clearly indicative of postsynaptic activation (rather than inhibition) of dopamine receptors. In line with this, we previously provided evidence for an increased dopamine level in the larger participant sample under 2 mg haloperidol based on fMRI activations [19]. The majority of that sample also took part in the current behavioural study, which allows us to evaluate the effects of increased dopamine on the emergence of explicit memory during incidental learning in the SRT task. This increase in dopamine level was further corroborated by re-analysing the fMRI data [19] for the current participant sample to confirm group differences in striatal activity observed in the larger participant sample [19].

## Materials and methods

### Participants

We used a double-blind, placebo-controlled between-group design with 54 participants in total which were randomly assigned to the haloperidol or control group as part of a larger study [19]. 42 of these participants (14 males, age 23.8±3.0 years; 20 haloperidol and 22 placebo) took part in the SRTT paradigm. The study was approved by the local ethics committee of the Hamburg medical association and written consent was given by each participant prior to the start of the study. Additionally, all participants were screened by a physician for previous or current physical or mental diseases, medication or drug use. All participants had normal or corrected-to-normal vision. The participants were informed about the study and about the potential risks and side effects of haloperidol. Moreover, the participants were instructed to restrain from caffeine, nicotine, and alcohol on the day of testing.

### Drug administration

Prior to drug administration, blood pressure and pulse were checked and the participants filled out a questionnaire assessing their current mood and potential adverse effects of the medication. Next, the participants received a tablet containing either a placebo or 2 mg of the D2-antagonist haloperidol (based on prior random assignment). Blood pressure and pulse measurements together with questionnaires on adverse medication effects and current mood were repeated 0.5h, 2h and 4.5h after drug/placebo administration. The SRT paradigm was preceded by the fMRI task [19] also described below and started approximately 4.5h after drug/placebo administration outside the fMRI scanner on a PC. At the end of the study all participants were asked to indicate the substance (haloperidol or placebo) they thought they had received and how certain they were of this guess.

### Stimuli

The coloured squares (0.4° x 0.4°; maximal distance 2.3°) were presented on a grey background controlled by a PC using the software “Presentation” (http://nbs.neuro-bs.com). Participants entered the responses by pressing buttons (3 buttons for each hand).

### SRTT design

**Fig 1:**
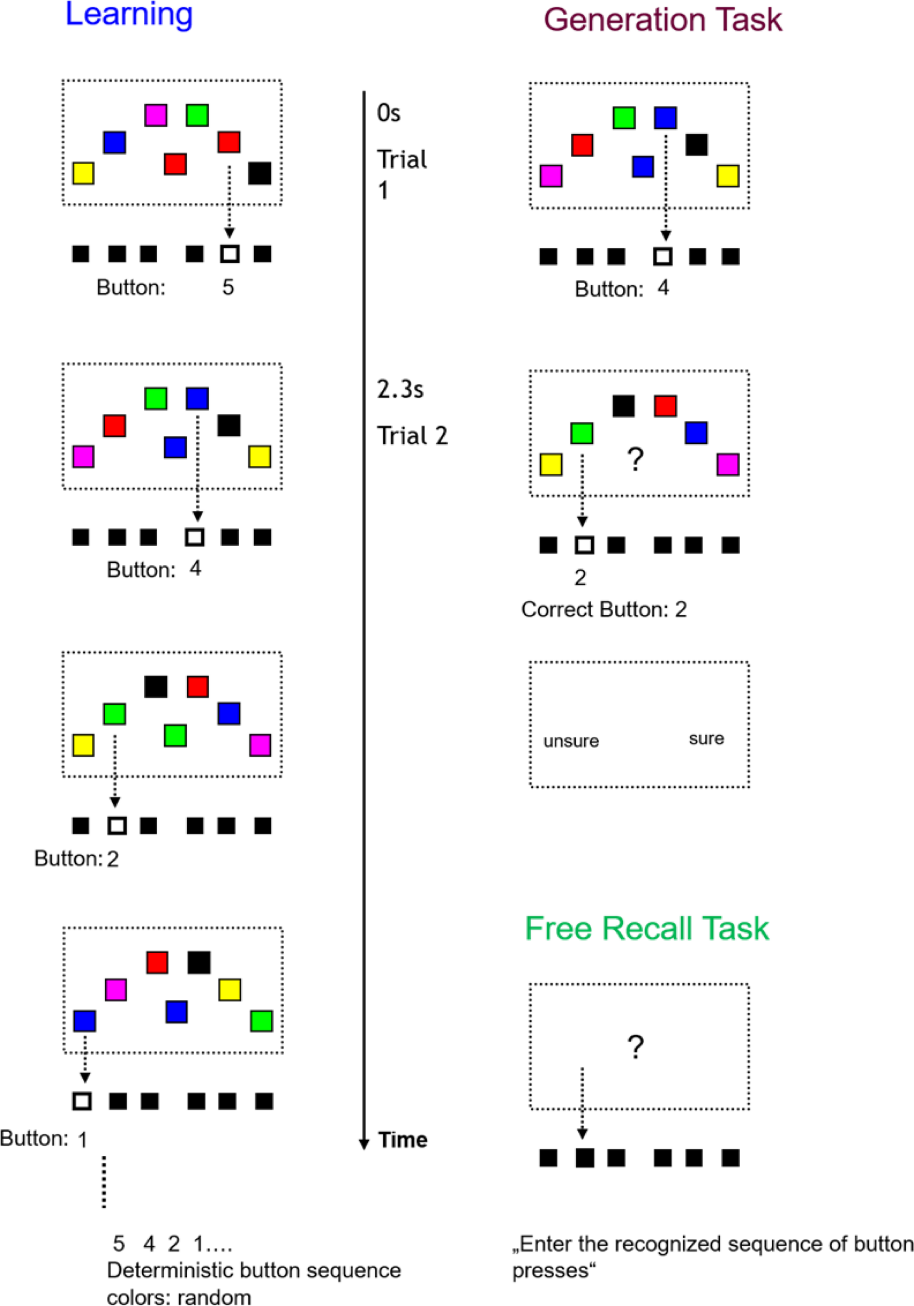
SRT task. In the learning period (left) 6 coloured squares (red, green, magenta, black, yellow and blue) were presented around the centre of the screen, the location of each square was assigned to a corresponding button. The target square in the middle simultaneously appeared at fixation and its colour indicated the button that should be pressed in the current trial (i.e., red in the centre indicates to press the finger assigned to the red square, in this case the second finger of the right hand). Importantly, in each trial the assignment of the colours to the response locations changed, which allowed the establishment of a motor sequence without a correlation between perceptual and response sequence. Explicit knowledge of this sequence was assessed with a generation task (right) where the volunteers were interrupted after each trial and asked to enter the colour of the next target and indicate the confidence about the answer. In the free recall task, the participants that reported the observation of a regular sequence were asked to enter the button presses without any visual cue.

In each of the 700 learning trials (ITI 300ms), volunteers were shown 6 colored squares (red, green, magenta, black, yellow, and blue) where the location of each square was assigned to the corresponding button and response finger (index, middle and ring finger of the left and right hand). The target square simultaneously appeared in the middle and its color indicated the button that should be pressed in the current trial (see Fig 1). Each stimulus arrangement was presented until a response was made for a maximum of two seconds. Importantly, the button responses were deterministic (finger 5, 4, 2, 1, 6, 3), but the colors of the squares changed unpredictably. Therefore, the embedded sequence was restricted to the motor domain and uncorrelated with target color.

The amount of explicit sequence knowledge was measured after the learning session using a generation task in combination with a confidence assessment [38]. The beginning of each generation trial was analogous to the training session but after the button assigned to the colored square was pressed, the participants were prompted by a question mark to guess about the next response (each transition in the sequence four times, 24 trials) and indicate their confidence by button press (low or high confidence). In addition, those participants that had previously stated to have noticed a regular sequence were asked to freely recall the remembered button sequence from the learning phase.

### Supplementary fMRI acquisition and analysis of picture onsets

While the SRTT was only measured behaviorally, we re-analyzed the fMRI data of the preceding unrelated recognition memory task [19] in the SRTT participants to estimate haloperidol effects on the striatal BOLD response to picture onsets. As one placebo participant had not been scanned due to technical problems, only 41 of the SRTT participants (20 haloperidol, 21 placebo) contributed to the fMRI data which are described in detail elsewhere [19]. Briefly, the participants saw in total 160 pictures (80 old and 80 new pictures) of outdoor street scenes presented for four seconds and had to indicate their confidence in recognizing these pictures from the encoding session on the previous day using button presses (see also [39,40]). The viewing of the pictures in the fMRI paradigm has previously been shown to evoke strong striatal activity [39]. Functional MR images were obtained on a 3T system Siemens Trio using single-shot echo-planar imaging with parallel imaging (GRAPPA, in-plane acceleration factor 2) [41] and simultaneous multi-slice acquisitions ("multiband", slice acceleration factor 2) as described in [42] (TR = 1.98s, TE = 26ms, flip angle = 70°, 64 axial slices, voxel size 2 x 2 x 2 mm^3^). The corresponding image reconstruction algorithm was provided by the University of Minnesota Center for Magnetic Resonance Research. In addition, an anatomical high-resolution T1-weighted image (TR = 2.3s, TE = 2.98ms, flip angle = 9°, 192 sagittal slices, voxel size 1 x 1 x 1 mm^3^) was acquired for each participant.

The data were pre-processed with SPM12 (http://www.fil.ion.ucl.ac.uk/spm/) using standard procedures (discarding the first five EPI images, correction for motion and for the interaction between motion and distortion, normalization of anatomical and EPI images to standard MNI space using DARTEL, and spatial smoothing of EPI images using a Gaussian kernel of 8 mm full width at half-maximum). Univariate single subject (first-level) and group (second-level) statistics were conducted using the general linear model implemented in SPM12. We set up a first-level model containing the onset regressors of all pictures by convolving the delta functions marking the trial onsets with the canonical hemodynamic response function to create an event-related picture onset regressor. This picture onset regressor was compared between groups by means of an independent samples t-test. The resulting activation maps were thresholded at p < 0.05 (family-wise error (FWE)-corrected for multiple comparisons at cluster level; cluster-forming threshold at voxel level: p < 0.001).

## Results

### Demographics

The haloperidol and the placebo group did not differ with regard to age, weight or sex (all p > .18). Moreover, there was no significant pattern of received and guessed substance (35% of haloperidol participants and 23% of the placebo participants guessed that they had received haloperidol, χ^2^(1, N = 42) = 0.77, p = .38) and no difference with regard to the certainty of guess (t(40) = 0.814, p = .42). There were no significant group differences in systolic blood pressure, diastolic blood pressure, or pulse relative to baseline. There were no significant group differences in reported side effects or in reported subjective feelings across measurements relative to baseline (16-item VAS grouped into the dimensions “alertness”, “calmness”, and “contentedness” [43]).

### SRTT paradigm

**Fig 2:**
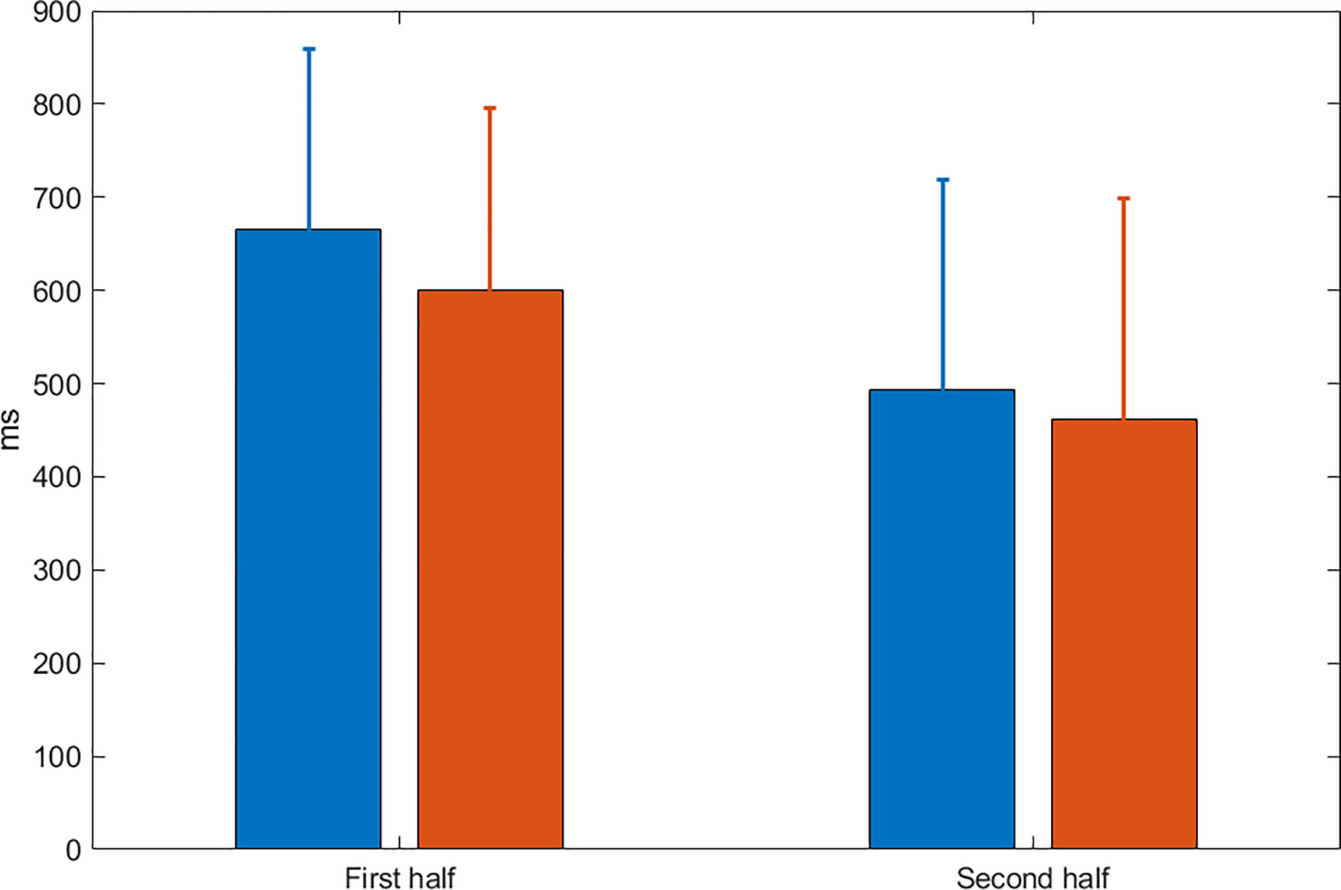
Mean RTs for each group compared between the first and second half of the learning period. No significant difference was observed between the haloperidol group (red) and the control group (blue). Error bars denote the standard deviation.

**Fig 3:**
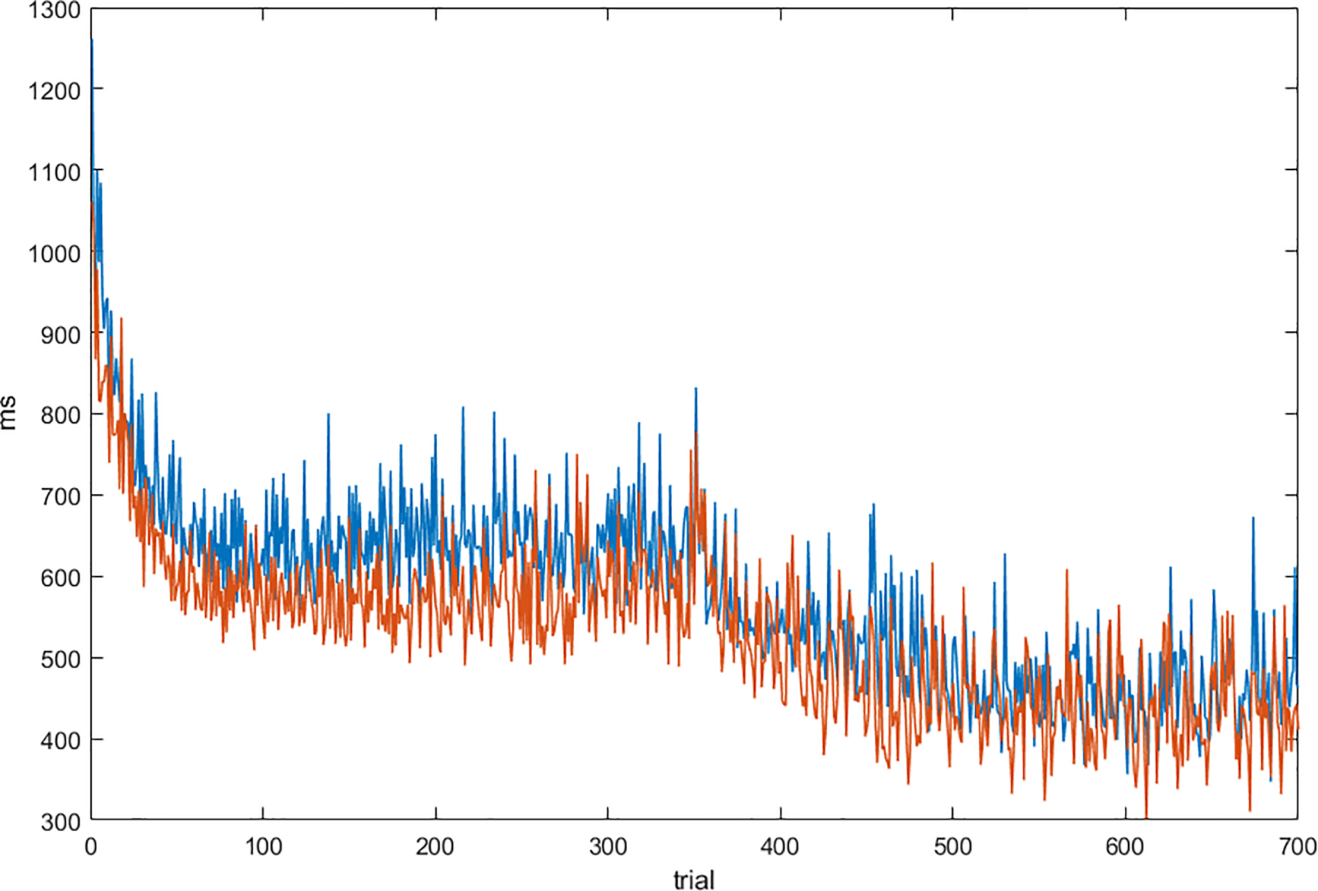
Mean RTs across learning trials for each group. No significant differences were observed between haloperidol (red) and control group (blue) tested across seven time bins in steps of 100 learning trials.

Learning was estimated by comparing reaction times (RTs) to target color presentation in the centre during the first and second half of the learning period. Mean RTs were calculated with respect to the onset of the correct response for each single input. A repeated measures ANOVA with the factors session (first/second half) and group (placebo/ haloperidol) revealed a general decrease of RTs across sessions (F(1,40) = 133.5, p < .0001) but no difference between groups (mean across learning: placebo group 1261ms; haloperidol group 1072ms F(1,40) = 1.6, n.s.) and no interaction effect (F(1,40) = 1.2, n.s.) indicating comparable RTs (see Fig 2).To address possible RT differences in more detail across the learning phase, we grouped the 700 trials into seven bins of 100 trials each and conducted between group t-tests for each time bin. In none of the seven time bins a significant difference in RTs was observed (p> 0.05; see Fig 3). Error rate during learning was low and not different between groups (placebo group: 4.2%; haloperidol group: 4.3%; t(40) = 0.12, n.s.).

**Fig 4:**
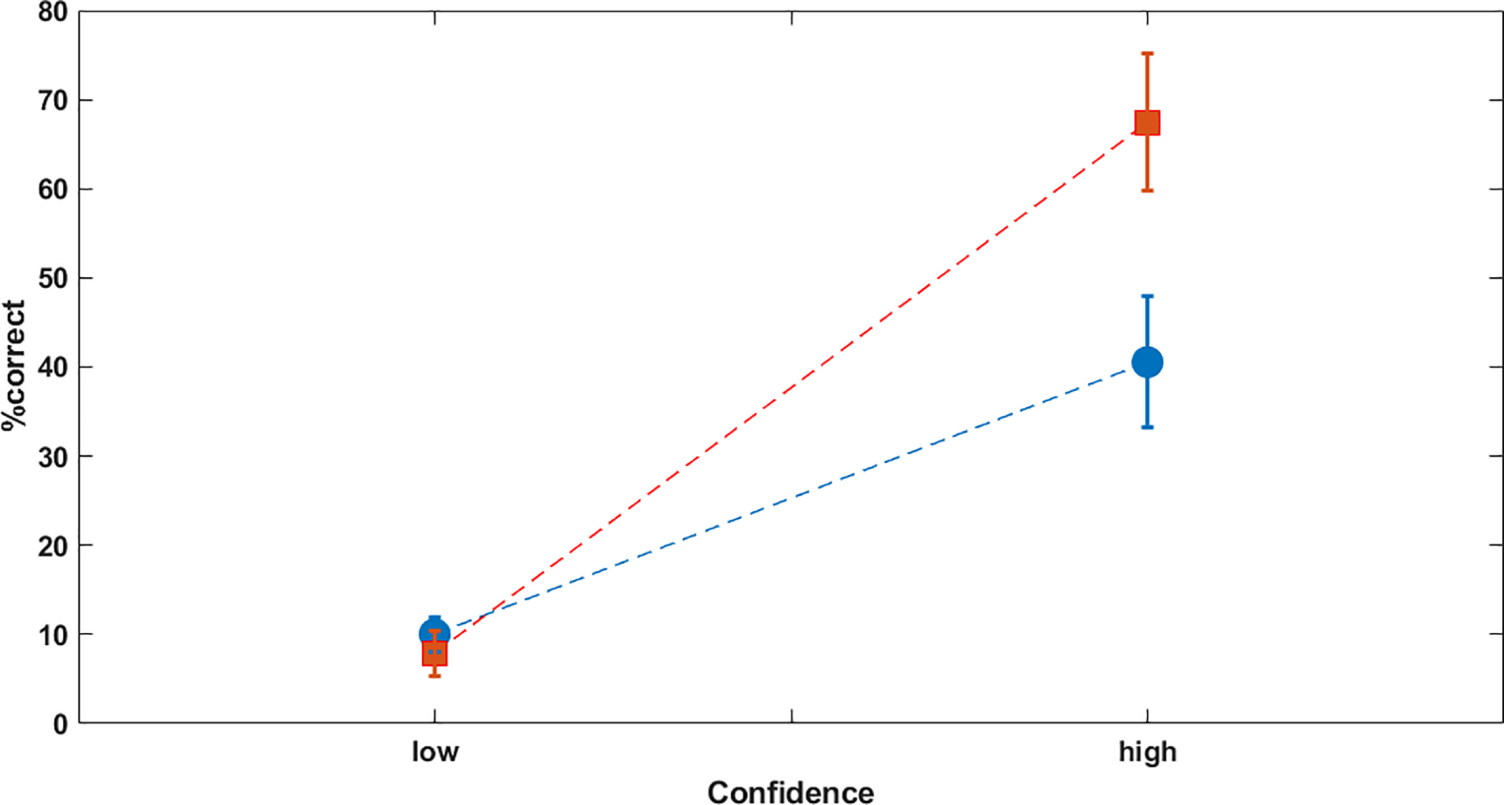
Generation task. Percentage of correct responses during the generation task separately for low and high confidence ratings (“unsure” or “sure” decision). The higher amount of high confident correct trials indicates significant enhanced explicit memory for the haloperidol group (red squares) compared to the control group (blue circles) (p<0.001).

In contrast, the generation task results indicated that explicit memory was significantly enhanced in the haloperidol group. The repeated measures ANOVA for the percent correct predictions of the following response (factors confidence (high vs. low) and group (placebo/ haloperidol)) revealed a main effect of group (F(1,40) = 8.0, p<0.01) with more correct responses in the haloperidol group, a main effect of confidence (F(1,40) = 11.5, p<0.001) and an interaction effect (F(1,40) = 5, p<0.05) indicating in particular higher accuracy in the haloperidol group for the high confidence responses (see Fig 4).

**Fig 5:**
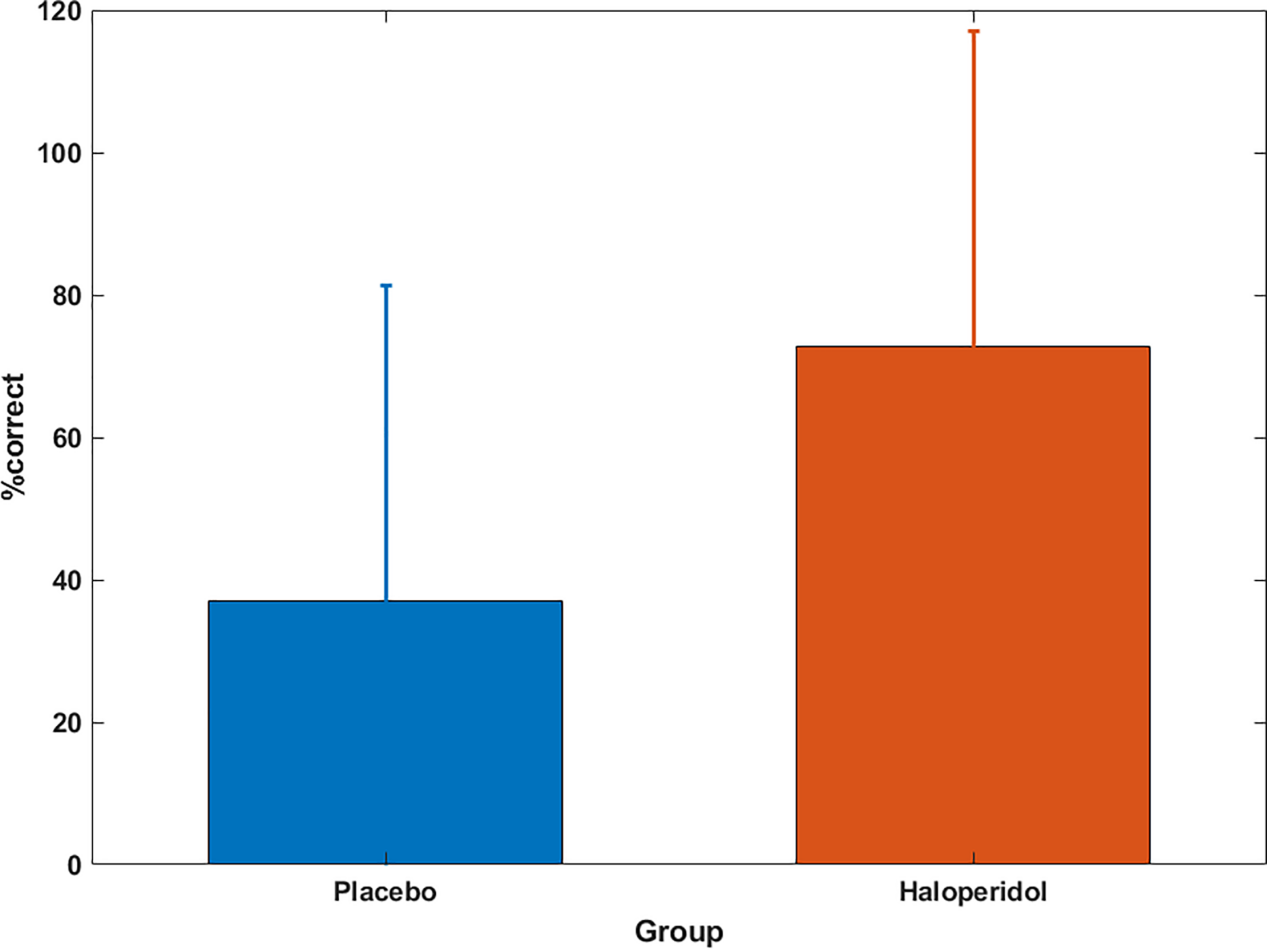
Free recall. Mean percentage of correctly recalled transitions in the free recall test for both groups. Within the haloperidol group the amount of explicit knowledge was nearly doubled compared to the placebo group (p = 0.01). Error bars denote the std.

Supporting these enhanced explicit memory results observed in the generation task, analysis of the free recall task data revealed that 70% of the haloperidol volunteers correctly recalled the complete sequence of six button presses, whereas in the placebo group only 36% showed complete explicit knowledge of the sequence (Fisher’s exact test, odds ratio = 4.1, p<0.0001). Similarly, significant group differences resulted when comparing the number of freely recalled correct transitions within the sequence (maximum of 6 transitions), where the haloperidol group had more explicitly recalled transitions (t(40) = 2.7, p = 0.01; Fig 5).

### Supplementary fMRI results: Striatal response to pictures

**Fig 6:**
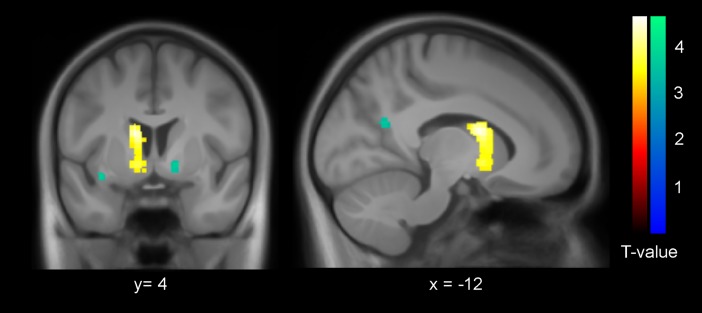
Higher striatal activation under haloperidol. The left striatum showed significantly higher activation in the haloperidol group (N = 20) compared to the placebo group (N = 21) across all picture trial onsets in the unrelated fMRI task preceding the SRTT (whole-brain FWE-corrected; warm colours). For visualization purposes, additional voxels significant only at the uncorrected threshold of P < 0.001 are displayed in cold colours.

As also observed for the full sample [19], the haloperidol group showed significantly higher activation in the left striatum (MNI -12/4/16; z = 4.11; 334 voxel) at p < 0.05 (FWE-corrected at cluster level; cluster-forming threshold at voxel level: p < 0.001; Fig 6) in response to picture onsets in the directly preceding task in the fMRI scanner. The activation cluster in the right striatum did not reach significance. No other significant activation differences across all trials were observed between the groups.

## Discussion

The present data provide evidence for a causal role of dopaminergic neurotransmission for the emergence of explicit memory during an incidental learning process. The low dose of the D2-antagonist haloperidol nearly doubled the amount of explicit sequence knowledge in the SRTT. Moreover, as also exemplified in more detail in [19], the fMRI data showed that the haloperidol group had overall significantly and selectively higher activation in the striatum in a task that strongly involved the striatum in previous fMRI studies [39,40].

Notably, optogenetically evoked phasic dopamine neuron activity in rats has been shown to lead to fMRI activity most prominently in the dorsal striatum [44,45].The increased activation in the haloperidol group across all trials primarily in the dorsal striatum thus clearly argues in favor of the view that acute low doses of D2-antagonists increase dopamine by blocking the presynaptic synthesis- and release-regulating autoreceptors [23–25,30], see also [19]. In addition to enhancement of dopamine concentration in the striatum due to haloperidol [29–31], it should be noted that some postsynaptic effects might be present even at low doses of D2 antagonists. For example, increased dopamine release combined with some postsynaptic D2 receptor blockade might decrease the ratio of D2 to D1 receptor activation [46,47]. Such an activation shift from D2 to D1 receptors might have contributed to the observed facilitation of explicit memory emergence in the haloperidol group, rather than increased dopamine release alone.

The beneficial effect of dopaminergic stimulation on explicit memory resonates well with the role of the striatum in the emergence of explicit memory during implicit learning as established in an earlier SRTT study [2]. This previous fMRI study demonstrated an increase in striatal activity in direct temporal relation to the individual time point of insight [2]. In particular, the striatal activity showed a clear increase over only a few learning trials directly preceding the learning trial in which the emergence of explicit knowledge was observed at the behavioral level of the individual participant. This temporally specific role of the striatum can be viewed as evidence for a distinct involvement of the striatum for the emergence of explicit memory which, as the current results suggest, is moreover dopamine-dependent. Striatal involvement in sequential learning was also demonstrated by studies using the classical version of the SRTT [48–52] and by one study reporting dopaminergic medication effects in patients with Parkinson’s disease in the SRTT [17]. Importantly, all the previous studies focused on the formation of implicit memory represented by RT changes across learning. In contrast, the present study demonstrated dopamine-dependent differences in the generation of explicit memory but a similar degree of implicit learning, as indicated by the absence of group differences in the general level of RTs and in the RT change across learning.

This differential effect of dopamine on explicit and implicit memory suggests that the development of explicit memory is not simply caused by a strengthening of the memory representation by implicit learning alone, as proposed in a theoretical model [1]. According to this model, an enhancement of explicit memory should not be possible without a correspondent enhancement of implicit memory. Instead, our data indicate that the emergence of explicit knowledge requires additional processes, such as restructuring stimulus representations on the basis of the ongoing evaluation of predictions, which selectively were facilitated by dopamine. Theoretical models assume an ongoing process that compares several parameters of the generated response in the actual trial with predictions about the processing, e.g. the speed or fluency that was necessary the produce the output [3,53]. These theories argue that during the course of implicit learning, the cognitive demands of the task decrease, since the gained implicit knowledge about the sequential regularities allows for the prediction of the upcoming stimulus or response. Due to this increased processing efficiency the evaluation of the stimulus and/or response, preparation can be accelerated. Thereby, a discrepancy develops between these experienced task demands (which decrease during implicit learning) and the expected task demands (which remain at the same level). This difference of the experienced and expected processing demands can be regarded as a prediction error, whose detection leads to an allocation of attention to the source of that prediction error. Subsequently, search processes are initiated, which result in the explicit detection of the hidden sequence of stimuli and the emergence of explicit knowledge about the structural relations. In line with these theoretical assumptions, it can be speculated that the low dose of haloperidol increased the sensitivity of the dopaminergic system responsible for the ongoing detection of outcome-related prediction error signals. This interpretation is in line with previous studies showing that dopaminergic manipulations affect striatal prediction errors in rewarding and aversive contexts [34,54–57] and with a pharmacological study on motor flexibility demonstrating that haloperidol increased the sensitivity to unpredictable events within an otherwise probabilistic environment, as reflected by a heightened response time indicating an increased evaluation of predicted and actual outcome [37].

Alternatively, increased dopaminergic stimulation might have resulted in increased explicit sequence memory by supporting the shift from model-free motor predictions to a more model-based sequence representation. According to this reinforcement learning view [58], motor behaviour in the SRTT is then initially ruled by a reflexive model-free strategy involving prediction error signals in the striatum [59]. However, at some point in the learning process the participant might be able to switch to a more goal-directed model-based strategy, which allows for stronger deliberate behavioural control and conscious access to the underlying sequence contingencies. This interpretation is supported by studies demonstrating that higher dopamine level was associated with more model-based behaviour in both healthy participants [60] and in patients with Parkinson’s disease [61]. Of interest, it has previously been argued that increased dopamine might facilitate the use of model-based strategies by disrupting model-free prediction error learning and thereby obliging participants to employ a more model-based strategy [60].

Thus, rather than improving striatal prediction error detection, the dopaminergic manipulation in the current study might have resulted in increased model-based learning and thereby evoked an enhanced explicit sequence representation. Dopaminergic effects on the balance between model-free and model-based behaviour have moreover been linked with striatal and prefrontal mechanisms [62], where the latter might constitute an important factor for awareness of the motor sequence in the current task.

In addition to the striatum and the prefrontal cortex, it should be noted that dopamine receptors are also present in MTL structures such as the hippocampus and the amygdala [63]. Increased dopaminergic stimulation of these regions might likewise contribute to the enhanced explicit memory observed under haloperidol. However, as a previous study suggested that only the perceptual (but not the motor) variant of the SRTT involves the hippocampus [64], this mechanism seems less likely for the dopamine-driven enhanced explicit memory in the motor domain observed here.

Finally, the increase in dopaminergic neurotransmission might have enhanced a more general motivational aspect of learning. In particular for reward related tasks a tight coupling between motivational state and tonic dopamine level is discussed [65]. However, such a general increase of the motivation state to learn would have affected the whole learning period and should be reflected in a profound response speed difference that was not observed in the present study, although the mean RTs in the haloperidol group are faster than in the placebo group but did not reached level of statistical significance.

Overall, the present results provide direct experimental evidence for a qualitative change in information processing as a prerequisite for the emergence of explicit memory during incidental learning in accord with several theoretical models [66–68] and a causal role of dopaminergic processing for this process. The increase in dopamine-related activity as evidenced by increased striatal activations under haloperidol resulted in a clear enhancement in the emergence of explicit memory. Finally, in contrast to the classical SRTT, the embedded sequence was restricted to the motor domain without any correlation to the visual stimuli. Therefore, the enhanced emergence of explicit sequence knowledge observed in the present modified version of the SRT task is specifically linked to increased dopamine-driven transformation of learned motor sequences into explicit memory, although similar effects mediated by dopaminergic receptors in the hippocampus might be expected for the perceptual SRTT [64].
